# Molecular Imaging of Metabolic Reprograming in Mutant IDH Cells

**DOI:** 10.3389/fonc.2016.00060

**Published:** 2016-03-14

**Authors:** Pavithra Viswanath, Myriam M. Chaumeil, Sabrina M. Ronen

**Affiliations:** ^1^Department of Radiology and Biomedical Imaging, University of California San Francisco, San Francisco, CA, USA

**Keywords:** mutant IDH1, metabolic reprograming, magnetic resonance spectroscopy, molecular imaging, cancer, 2-hydroxyglutarate, low-grade gliomas

## Abstract

Mutations in the metabolic enzyme isocitrate dehydrogenase (IDH) have recently been identified as drivers in the development of several tumor types. Most notably, cytosolic IDH1 is mutated in 70–90% of low-grade gliomas and upgraded glioblastomas, and mitochondrial IDH2 is mutated in ~20% of acute myeloid leukemia cases. Wild-type IDH catalyzes the interconversion of isocitrate to α-ketoglutarate (α-KG). Mutations in the enzyme lead to loss of wild-type enzymatic activity and a neomorphic activity that converts α-KG to 2-hydroxyglutarate (2-HG). In turn, 2-HG, which has been termed an “oncometabolite,” inhibits key α-KG-dependent enzymes, resulting in alterations of the cellular epigenetic profile and, subsequently, inhibition of differentiation and initiation of tumorigenesis. In addition, it is now clear that the IDH mutation also induces a broad metabolic reprograming that extends beyond 2-HG production, and this reprograming often differs from what has been previously reported in other cancer types. In this review, we will discuss in detail what is known to date about the metabolic reprograming of mutant IDH cells, and how this reprograming has been investigated using molecular metabolic imaging. We will describe how metabolic imaging has helped shed light on the basic biology of mutant IDH cells, and how this information can be leveraged to identify new therapeutic targets and to develop new clinically translatable imaging methods to detect and monitor mutant IDH tumors *in vivo*.

## Introduction

Altered cellular metabolism is a feature of malignant cancer cells (1–4). In the 1920s, Warburg described the elevated conversion of glucose into lactate, which occurs in tumor cells even under normoxia (Warburg effect) (5). Contrary to Warburg’s hypothesis that held defective mitochondrial function responsible for aerobic glycolysis, it is now understood that tumor cells actively reprogram cellular metabolism to support tumor growth and metastasis (6–8). This increased glucose consumption and glycolytic flux contribute to acidification of the microenvironment, likely facilitating metastasis (9). Furthermore, glycolytic intermediates are used for anabolic reactions leading to nucleotide, phospholipid, and amino acid biosynthesis, providing the building blocks required for cell proliferation (7, 8, 10). Additionally, glutaminolysis provides the anaplerotic flux to replenish TCA cycle intermediates depleted for biosynthetic purposes and generates NADPH required for redox homeostasis and lipid synthesis (11–13). Choline metabolism is also modulated to provide precursors for membrane biosynthesis (14).

To date, the emerging paradigm recognizes that oncogene and tumor suppressor signaling pathways are responsible for the deregulation of metabolic pathways in cancer (15–22). Mutations in the PI3K and LKB1–AMPK signaling pathways, Myc and Ras oncogenes, and the tumor suppressor p53 all reprogram metabolism (16, 23–34). However, the discovery of tumors with gain-of-function mutations in metabolic enzymes provides strong evidence that altered metabolism can also result from mutations in metabolic enzymes. This is particularly true for tumors with mutations in the cytosolic or mitochondrial forms of isocitrate dehydrogenase (IDH1 and IDH2, respectively) (19, 35, 36).

Mutations in IDH1 were first described in a whole-genome sequence analysis of glioblastoma patients (37). Subsequent studies confirmed the presence of IDH mutations in 70–90% of low-grade glioma and secondary glioblastoma, in ~20% of acute myeloid leukemia, and in intrahepatic cholangiocarcinoma, chondrosarcoma, and melanoma (36, 38, 39). The IDH1 mutation is one of the earliest known genetic events in low-grade gliomas, and it is thought to be a “driver” mutation for tumorigenesis (40). Discovery of the IDH1 mutation has also led to a molecular (rather than histological) classification of gliomas (41). Presence of the IDH1 mutation in this new classification is associated with a more favorable prognosis compared to tumors with wild-type IDH1 (42). The reasons for this better prognosis remain to be determined, but different cellular metabolism could be a contributing factor.

From a metabolic perspective, mutations in IDH1 and IDH2 lead not only to the loss of wild-type enzyme activity [interconversion of isocitrate to α-ketoglutarate (α-KG)] but also to a gain-of-function that results in the conversion of α-KG to the “oncometabolite” 2-hydroxyglutarate (2-HG) (43). 2-HG is a competitive inhibitor of multiple α-KG-dependent dioxygenases, such as the prolyl hydroxylases, the Jumonji C family of histone demethylases, and the TET family of DNA hydroxylases (44). As a result, IDH1/2 mutant cells undergo extensive epigenetic modifications that ultimately result in tumorigenesis (45–48).

Among other changes, the IDH mutation leads to alterations in cellular metabolism extending beyond 2-HG production. Interestingly, many of these changes differ from those observed in other, non-IDH mutated, cancer cells. To date, the metabolic characterization of mutant IDH cells has been carried out using either mass spectrometry (MS) or magnetic resonance spectroscopy (MRS) (49). MS has the advantage of exquisite sensitivity (as low as picomolar) yielding a wealth of information on a wide range of cellular metabolites. However, with some exceptions (e.g., acute myeloid leukemia), MS requires the destruction of cell/tissue sample; and hence, clinical translation is limited. MRS can only detect metabolites above 0.1–1 mM and *in vivo* spectra at clinical field strengths cannot resolve closely resonating metabolites. Nonetheless, MRS can be used as a translational, non-invasive modality to detect and quantify metabolites in cells and *in vivo* in animals and patients. ^1^H- and ^31^P-MRS can be used to quantify steady-state metabolite levels, whereas ^13^C- and hyperpolarized ^13^C-MRS can be used to monitor metabolic fluxes (50–55).

In this review, we will discuss what is known about the metabolic reprograming of mutant IDH cells from a molecular imaging perspective. We will begin by reviewing the various MRS approaches that have been applied to image 2-HG. This will be followed by a comprehensive discussion of metabolic alterations in mutant IDH tumors and the imaging methods used to investigate these changes. We will describe how molecular imaging has helped shed light on the basic biology of mutant IDH cells and will address how this knowledge could serve to identify new therapeutic targets and novel methods for imaging mutant IDH tumors in the clinic.

## Imaging 2-HG and 2-HG Production

### *Ex Vivo* Measurement of 2-HG Levels

**Figure 1:**
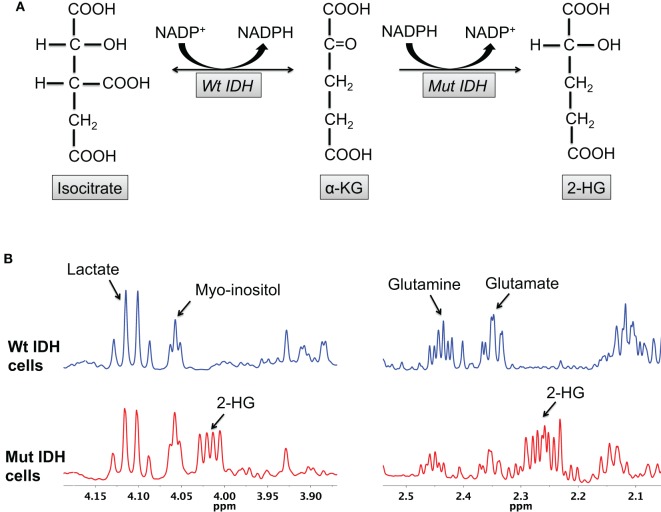
**2-HG production is characteristic of IDH mutant glioma cells**. **(A)** The wild-type IDH enzyme catalyzes the interconversion of isocitrate and α-KG, whereas the mutant enzyme irreversibly converts α-KG to 2-HG. **(B)** 2-HG peaks (2.25 and 4 ppm) can be observed in the spectra of mutant, but not wild-type, IDH cells.

The most obvious metabolic change in IDH mutant cells is the production of 2-HG (Figure 1). Using MS, Dang et al. reported elevated levels of 2-HG (5–35 μmol/g tissue) in patient glioma tissues (43). Gross et al., again using MS, reported elevated 2-HG levels (~10,000 ng/2 × 10^6^ cells) in extracts from patients with IDH1/2 mutant acute myeloid leukemia (38). Elkhaled et al. used ^1^H high-resolution magic-angle spinning spectroscopy (HRMAS) to quantify 2-HG levels in patients with low-grade glioma (56). 2-HG levels correlated with the IDH1 mutation determined by immunohistochemistry with 86% concordance. Interestingly, 2-HG levels across tumor samples of different grades correlated positively with increased cellularity and mitotic density on histopathology, suggesting that the amount of 2-HG per cell remained unchanged during malignant transformation. This finding is consistent with the role of mutant IDH1 as a driver mutation essential for initiating tumorigenesis (40). Kalinina et al. also analyzed tumor biopsy samples from low-grade glioma patients using two-dimensional (2D) correlation spectroscopy (COSY) (57). In a randomized blinded analysis of 45 glioma samples, spectroscopic analysis was successful in quantifying the 2-HG cross-peaks in IDH mutant tissues with 97.8% accuracy.

### *In Vivo* Measurement of 2-HG Levels

Although 2-HG levels are relatively high in IDH1 mutant tumors (5–35 mM), *in vivo* detection using ^1^H-MRS is hampered by the presence of overlapping resonances from glutamate and glutamine in the 2–3 ppm region of the spectrum. Strategies to enable proper 2-HG quantification therefore need to be implemented, either at acquisition or at postprocessing.

Two studies validated a single-voxel ^1^H-MR double-echo Point RESolved Spectroscopy (PRESS) sequence to estimate 2-HG levels in mutant IDH1 tumor patients (58, 59). Pope et al. evaluated 27 patients with glial tumors using a dedicated LC-model postprocessing analysis to measure 2-HG in tumor voxels. They found significantly elevated 2-HG levels in IDH mutant tumors compared to wild-type tumors and correlated the 2-HG levels with values measured by MS (58). Choi et al. examined ^1^H-MRS data from 30 glioma patients in a manner blinded to IDH mutational status (59). In this study, in addition to postprocessing dedicated to fitting the data, the authors also carefully optimized the acquisition echo time to minimize the overlap between 2-HG and glutamate/glutamine resonances. In every case where 2-HG was detected by MRS, the sample showed the presence of an IDH1/2 mutation. Conversely, the absence of a 2-HG signal was associated with IDH wild-type status. In a third study, Andronesi et al. used a more complex 2D-COSY MRS method to detect 2-HG in mutant IDH1 glioma patients and in *ex vivo* biopsy samples (60). Use of the 2D acquisition method could prevent false-positive detection of 2-HG that might result from improper fitting of 1D MR spectra and the spectral proximity of 2-HG to glutamate/glutamine. However, the acquisition time for 2D data is significantly longer than 1D method and thus potentially more challenging to implement in the clinic.

### *In Vivo* Measurement of 2-HG Production

**Figure 2:**
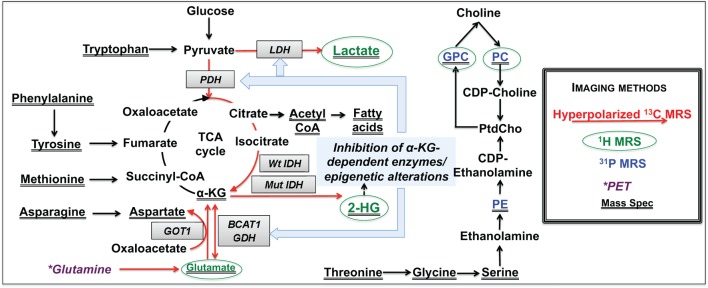
**Overview of metabolic reprograming in IDH mutant glioma cells**. Metabolic alterations detectable by non-invasive MRS are highlighted in red for hyperpolarized ^13^C-MRS, green for ^1^H-MRS, and blue for ^31^P-MRS. Changes detected by mass spectrometry are underlined, and those that can potentially be detected by PET imaging are shown in dark purple.

^13^C-MRS has been used extensively, especially in the preclinical arena, to inform on real-time metabolic fluxes by probing the fate of exogenous ^13^C-labeled substrates (61). However, ^13^C-MRS lacks sensitivity and therefore requires relatively long acquisition times to achieve an adequate signal-to-noise ratio (SNR), limiting its implementation *in vivo*. The recent development of dissolution dynamic nuclear polarization (DNP) can overcome this limitation. Using dissolution DNP, ^13^C-labeled compounds can be hyperpolarized, dissolved into solution, injected into the sample (or subject), and be rapidly detected by MRS with a 10,000- to 50,000-fold increase in SNR compared to thermally polarized compounds (62, 63). ^13^C-MRS of hyperpolarized compounds has been used to monitor enzymatic activities in solution, cells, and *in vivo* (51, 64–66). Using this technology, our laboratory designed and validated a new DNP probe, hyperpolarized [1-^13^C]-α-KG, for non-invasive ^13^C-MRS imaging of 2-HG synthesis. We showed that, following injection of hyperpolarized [1-^13^C]-α-KG, the production of hyperpolarized [1-^13^C]-2-HG could be detected in lysates and in orthotopic mutant IDH1 tumors in rodents, but not in their wild-type counterparts (Figure 2) (67). By providing dynamic information with regard to the metabolic fate of hyperpolarized [1-^13^C]-α-KG, this approach provides complementary information to ^1^H-MRS, which detects steady-state levels of 2-HG. As such, the hyperpolarized method can inform in real-time on the presence of active mutant IDH1 and on potential inhibition of mutant IDH1 by novel therapies.

### Metabolic Precursors of 2-HG

Although thermal equilibrium ^13^C-MRS is not readily translatable, it can be used to monitor metabolic fluxes in the preclinical setting and has been used to identify the metabolic precursors of 2-HG. Dang et al. demonstrated that glutamine is the major precursor of 2-HG (43). However, we and others have demonstrated that glucose also contributes to 2-HG synthesis. In a study by Pichumani et al., mutant IDH1 glioma patients received an infusion of [U-^13^C]-glucose during surgical tumor resection (68). *Ex vivo* ^13^C-MRS on biopsy extracts revealed ^13^C-labeling in 2-HG, indicating that glucose contributes to 2-HG production. In our laboratory, we incubated two IDH1 mutant glioma models with [1-^13^C]-glucose and [3-^13^C]-glutamine and analyzed the proportion of ^13^C-labeled 2-HG derived from each precursor. We found that ~20% of 2-HG was derived from glucose and ~80% from glutamine (69). These findings have therapeutic implications since inhibiting glutaminase (the enzyme that converts glutamine to glutamate, the precursor of α-KG and 2-HG) has been explored as a therapeutic target for IDH mutant cells, without considering that glucose could serve as an alternate source of 2-HG (70, 71).

## Imaging Metabolic Reprograming in IDH Mutant Cells

Although the most obvious metabolic change in IDH mutant cells is the production of 2-HG, a number of studies indicate that IDH mutant cells undergo broader metabolic reprograming. Reitman et al. conducted an MS-based metabolomic analysis of oligodendroglioma cells engineered to express wild-type or mutant IDH1 and IDH2 (72). Mutant IDH1/2 cells showed significantly increased levels of several amino acids, such as glycine, serine, threonine, asparagine, phenylalanine, tyrosine, tryptophan, and methionine (Figure 2). Glycerophosphocholine (GPC) levels were also higher, whereas glutamate, aspartate, and *N*-acetylated amino acid levels were reduced in IDH mutant cells compared to wild-type (Figure 2). Ohka et al. also carried out an MS analysis of wild-type or mutant IDH patient glioma tissues and reported a significant decrease in the levels of *N*-acetylated amino acids and glutamate (73). Additionally, both studies reported no change in glycolytic or pentose phosphate pathway intermediates in IDH mutant cells. We used high-resolution ^1^H-MRS to compare the metabolome of U87 cells expressing wild-type or mutant IDH1 and the metabolome of normal human astrocytes (NHA) expressing wild-type or mutant IDH1 (74). In line with MS observations, we found that the ^1^H-MRS-detectable steady-state levels of intracellular lactate, glutamate, and phosphocholine (PC) were significantly reduced in IDH1 mutant cells relative to wild-type, and GPC levels were higher (Figure 2). Collectively, these studies demonstrated that mutant IDH cells broadly reprogram their metabolism and laid the foundation for more in-depth investigations, as reviewed below.

### Aerobic Glycolysis

In our ^1^H-MRS study, we observed a reduction in intracellular lactate levels in IDH1 mutant glioma cells (Figure 2), suggesting that their metabolic reprograming could differ from other types of cancer cells (74). Chesnelong et al. confirmed this hypothesis and demonstrated that expression of lactate dehydrogenase A (LDHA), which catalyzes the production of lactate from pyruvate, was reduced in IDH mutant patient-derived glioma tissues compared to IDH wild-type glioblastoma that display elevated lactate production (75, 76). Importantly, LDHA silencing was mediated by increased promoter methylation consistent with the hypermethylator phenotype of IDH mutant cells.

In an effort to develop a complementary and clinically relevant imaging method for probing mutant IDH1-associated LDHA silencing, we recently investigated the fate of hyperpolarized [1-^13^C]-pyruvate in the BT142 patient-derived mutant IDH1 model *in vivo*. We found that hyperpolarized [1-^13^C]-lactate produced from hyperpolarized [1-^13^C]-pyruvate was comparable between mutant IDH1 tumors and normal brain in the BT142 model, in contrast to wild-type IDH1 glioma models, wherein hyperpolarized [1-^13^C]-lactate is significantly higher than in normal brain (77).

### Glutamate Metabolism and TCA Cycle

Glutamate levels are reduced in IDH mutant cells compared to wild-type (72–74). Furthermore, using ^1^H-MRS, Choi et al. showed that mutant IDH1 tumors showed reduced glutamate levels compared to normal brain, indicating that reduced glutamate could serve as a biomarker of mutant IDH1 tumors (78). In an effort to understand and image the metabolic alterations leading to glutamate reduction, several studies have been performed, each investigating a different step in glutamate production.

Branched chain amino acid (BCAA) transferase (BCAT) 1 and 2 catalyze the transfer of an amino group from BCAA to α-KG, resulting in the production of glutamate and α-keto acid. Tonjes et al. reported that BCAT1 expression was significantly reduced in IDH mutant cells (79). To image this reprograming, we expanded on our previous study (67) and used hyperpolarized [1-^13^C]-α-KG to monitor hyperpolarized [1-^13^C]-glutamate production in mutant IDH1 tumors (80). We showed that conversion of hyperpolarized [1-^13^C]-α-KG to glutamate was reduced in mutant IDH1 tumors compared to wild-type, in line with decreased BCAT1. In addition, we observed decreased expression of aspartate transaminase (GOT1) and glutamate dehydrogenase (GDH), two other enzymes catalyzing α-KG to glutamate metabolism, suggesting additional metabolic reprograming associated with the IDH1 mutation (Figure 2). BCAT1 and GOT1 promoter methylation is higher in mutant IDH cells, providing a likely mechanistic link between the IDH1 mutations and reduced α-KG to glutamate conversion (46, 79).

When considering the hyperpolarized approach for imaging glutamate production, our studies monitoring the fate of hyperpolarized [1-^13^C]-α-KG used pulse sequences optimized for the detection of only one metabolite: 2-HG or glutamate. However, further optimization of pulse sequences for detection of *both* 2-HG and glutamate could provide a molecular imaging approach that would simultaneously image IDH mutational status and the metabolic reprograming specifically associated with the mutation.

In an effort to consider additional metabolic alterations that could lead to a drop in glutamate levels, we used thermal equilibrium ^13^C-MRS to probe upstream metabolic precursors of glutamate (69). We found that there was a significant reduction in ^13^C-labeled-glutamate derived from [1-^13^C]-glucose in IDH mutant cells compared to wild-type resulting from lower PDH activity (69). Further mechanistic studies revealed that PDH activity was reduced due to increased inhibitory phosphorylation mediated by elevated expression of pyruvate dehydrogenase kinase 3 (PDK3), downstream of mutant IDH-driven stabilization of hypoxia inducible factor-1α (44, 81, 82). Importantly, treatment of IDH mutant cells with the PDH agonist dichloroacetate (DCA), not only reversed the metabolic changes induced by the IDH mutation but also abrogated the clonogenic potential of IDH1 mutant cells (69). This suggests that reprograming of PDH activity is essential for tumorigenesis of mutant IDH cells and that PDK inhibitors/PDH agonists deserve further investigation as potential therapeutic targets for low-grade gliomas. From an imaging perspective, we also demonstrated that PDH-mediated conversion of hyperpolarized [2-^13^C]-pyruvate to hyperpolarized [5-^13^C]-glutamate could be used to monitor the mutant IDH-driven drop in PDH activity in cells (Figure 2) (69), with potential *in vivo* implementation (83).

### Glutamine Metabolism

As with glycolysis, the reprograming of glutamine metabolism differs in mutant IDH cells compared to other cancer cells. Cancer cells can use a reductive pathway of glutamine metabolism in which wild-type IDH carboxylates α-KG to isocitrate (84–86). Subsequent conversion of isocitrate to citrate and of citrate to acetyl CoA contributes to fatty acid synthesis (86, 87). However, mutant IDH1 and IDH2 cannot catalyze reductive carboxylation (88), and IDH1 mutant cells show reduced metabolism of glutamine to citrate and acetyl CoA, resulting in altered fatty acid biosynthesis (89, 90). In addition, as mentioned, glutamine is the primary precursor of 2-HG. Imaging the fate of glutamine could therefore provide a useful complement to other metabolic imaging methods for detecting IDH status.

Infusion of human glioma patients with [U-^13^C]-glutamine prior to surgery, followed by ^13^C-MR analysis of metabolites extracted from tumor tissue has been used to estimate glutamine metabolism in brain tumors (91), but it is challenging to implement *in vivo*. ^13^C-MRS for probing the conversion of hyperpolarized [5-^13^C]-glutamine to hyperpolarized [5-^13^C]-glutamate has been reported in liver and prostate cancer cells (92, 93) and in rat liver tumor *in vivo* (93) and could potentially be used to characterize mutant IDH tumors. Interestingly, Venneti et al. described positron emission tomography (PET) imaging of glutamine metabolism using the glutamine analog 4-^18^F-(2S,4R)-fluoroglutamine (^18^F-FGln) in wild-type gliomas (94). They showed uptake of ^18^F-FGln in mouse xenografts *in vivo*, reduced ^18^F-FGln uptake in response to temozolomide treatment, and clinical translatability to glioma patients. Further research is needed to assess the value of this approach to mutant IDH gliomas.

### Phospholipid Metabolism

Mass spectrometry and ^1^H-MRS studies have shown a drop in PC and increase in GPC in mutant IDH1 cells compared to wild-type (Figure 2). Esmaeili et al. recently used ^31^P-MRS to further assess phospholipid metabolism in glioma rodent xenografts and in human biopsy samples (95). They confirmed that IDH mutant tumors showed higher levels of GPC and also found lower levels of phosphoethanolamine (PE) (Figure 2). Furthermore, ratios of GPC:PE, PC:PE, GPC:glycerophosphoethanolamine (GPE), and (PC + PE:GPC + GPE) were higher in IDH mutant tumors relative to wild-type (95). Further studies are needed to understand the significance of these findings and possible correlations between choline-containing metabolites and IDH status. Nonetheless, ^31^P-MRS could prove useful for non-invasive imaging of IDH mutant tumors.

## Conclusion

Cancer cells actively reprogram their metabolism to sustain and drive increased cell proliferation. At the preclinical level, metabolic imaging allows visualization of biochemical pathways promoting a better understanding of the physiological mechanisms of tumorigenesis. It also serves to identify new therapeutic targets. Further studies are needed to fully elucidate the wide range of metabolic changes occurring in mutant IDH cells. Nonetheless, the unique features of glucose, glutamine, and lipid metabolism identified to date can already be exploited for molecular imaging of mutant IDH tumors. Clinical deployment of these imaging methods could provide a useful complement to anatomical imaging methods and aid in tumor detection and monitoring of treatment response.

## Author Contributions

All authors listed, have made substantial, direct, and intellectual contribution to the work, and approved it for publication.

## Conflict of Interest Statement

The authors declare that the research was conducted in the absence of any commercial or financial relationships that could be construed as a potential conflict of interest.
